# Genomic alterations in neuroendocrine prostate cancer: A systematic review and meta‐analysis

**DOI:** 10.1002/bco2.212

**Published:** 2023-01-02

**Authors:** Junru Chen, Mingchen Shi, Stephen Yiu Chuen Choi, Yu Wang, Dong Lin, Hao Zeng, Yuzhuo Wang

**Affiliations:** ^1^ Department of Urology, Institute of Urology, West China Hospital Sichuan University Chengdu Sichuan China; ^2^ Vancouver Prostate Centre Vancouver BC Canada; ^3^ Department of Urologic Sciences, Faculty of Medicine University of British Columbia Vancouver BC Canada; ^4^ Department of Experimental Therapeutics BC Cancer Agency Vancouver BC Canada

**Keywords:** copy number alteration, genomic alteration, mutation, neuroendocrine prostate cancer, prevalence

## Abstract

**Background:**

Neuroendocrine prostate cancer (NEPC) is a lethal subtype of prostate cancer. We performed a systematic review and meta‐analysis to evaluate the prevalence of genomic alterations in NEPC and better understand its molecular features to potentially inform precision medicine.

**Methods:**

EMBASE, PubMed, and Cochrane Central Register of Controlled Trials databases were searched for eligible studies until March 2022. Study qualities were assessed using the Q‐genie tool. The prevalence of gene mutations and copy number alterations (CNAs) were extracted, and meta‐analysis was performed using R Studio with *meta* package.

**Results:**

A total of 14 studies with 449 NEPC patients were included in this meta‐analysis. The most frequently mutated gene in NEPC was *TP53* (49.8%), and the prevalence of deleterious mutations in *ATM/BRCA* was 16.8%. Common CNAs in NEPC included *RB1* loss (58.3%), *TP53* loss (42.8%), *PTEN* loss (37.0%), *AURKA* amplification (28.2%), and *MYCN* amplification (22.9%). *RB1/TP53* alterations and concurrent *RB1* and *TP53* alterations were remarkably common in NEPC, with a prevalence of 83.8% and 43.9%, respectively. Comparative analyses indicated that the prevalence of (concurrent) *RB1/TP53* alterations was significantly higher in de novo NEPC than in treatment‐emergent NEPC (t‐NEPC).

**Conclusions:**

This study presents the comprehensive prevalence of common genomic alterations and potentially actionable targets in NEPC and reveals the genomic differences between de novo NEPC and t‐NEPC. Our findings highlight the importance of genomic testing in patients for precision medicine and provide insights into future studies exploring different NEPC subtypes.

## 1 INTRODUCTION

Neuroendocrine prostate cancer (NEPC) is an aggressive subtype of prostate cancer (PCa) with a median overall survival of less than 1 year.^1^, ^2^ Although de novo NEPC is rare, accounting for less than 1% of all PCa cases, treatment‐emergent NEPC (t‐NEPC) can be detected in approximately 20% of metastatic castration‐resistant prostate cancer (mCRPC) in response to hormonal therapies.^3^, ^4^ Moreover, it has been suggested that the incidence of t‐NEPC may be increasing as a result of the widespread use of potent androgen receptor pathway inhibitors such as abiraterone, enzalutamide, and apalutamide.^5^ Unfortunately, few effective treatment options are available for NEPC patients due to a poor understanding of the disease. Given the similarity between NEPC and small cell lung cancer, platinum‐based chemotherapy, the first‐line treatment for small cell lung cancer, is commonly used to treat NEPC patients. However, it has only limited efficacy.^6^, ^7^ Thus, insights into the molecular determinants of NEPC development and identification of potential therapeutic targets are critically and urgently needed to better manage this lethal disease.

Over the past decade, the accelerating development of genomic testing technologies has enabled us to dig deeper into cancer biology, facilitating precision oncology. Using these technologies, recent studies have revealed several genomic alterations enriched in NEPC, including *RB1* loss, *TP53* mutation or deletion, and *AURKA/MYCN* amplification.^8^, ^9^, ^10^ In preclinical models, inactivation of Rb1 and Trp53 in mouse prostate adenocarcinoma can drive resistance to antiandrogen therapy and promote neuroendocrine transdifferentiation.^11^, ^12^ Collectively, these data suggest that genomic alterations play a critical role in NEPC pathogenesis. However, due to frequent misdiagnoses in tumours with mixed histology and limited metastatic tumour biopsy samples, most previous studies only enrolled a small number of NEPC patients, resulting in the reported prevalence of genomic alterations being highly variable. Moreover, genomic heterogeneity between different subtypes, such as de novo NEPC and t‐NEPC, is yet to be elucidated.

Here, we report the first systematic review of genomic alterations in NEPC and perform a prevalence meta‐analysis of gene mutations and copy number alterations (CNAs).

## 2 MATERIAL AND METHODS

### 2.1 Search strategy and eligibility criteria

This study was conducted following the Preferred Reporting Items for Systematic Review and Meta‐Analyses (PRISMA) guidelines.^13^ The study protocol is registered at the International Prospective Register of Systematic Reviews (PROSPERO, registration number: CRD42022310483). PubMed, EMBASE, and Cochrane Central Register of Controlled Trials databases were searched until March 31, 2022. The full search strategy is available in the registered protocol. Studies reporting the prevalence of genomic alterations in NEPC were eligible for this meta‐analysis. The exclusion criteria were (1) studies focusing on histologies other than NEPC; (2) case reports, reviews, comments, experimental studies, and conference abstracts; (3) non‐English studies.

### 2.2 Study selection and data extraction

Two reviewers (JRC and MCS) independently screened and double‐checked the titles and abstracts of the identified studies. Full texts of potentially eligible studies were retrieved and evaluated for final inclusion. Data extraction was performed by the two authors (JRC and MCS) independently. The following data were extracted from each included study: author, publication year, number of patients, age, NEPC type, number of samples, sample location, DNA source, sequencing methodology, and prevalence of genomic alterations. Considering the possibility of overlapping populations in different studies, only the data first reported were extracted. Any discrepancies were resolved by consensus.

### 2.3 Quality assessment

The Q‐genie tool was used to assess the quality of the included studies.^14^ The following nine domains were evaluated by 11 questions: Study rationale, sample selection, exposure, outcome, sources of bias, statistical plan, statistical method, testing of assumptions, and results interpretation. Each question was scored from 1 to 7. For studies with control groups, scores ≤35 indicate poor quality, 35–45 indicate moderate quality, and >45 indicate good quality. For studies without control groups, scores ≤32 indicate poor quality, 32–40 indicate moderate quality, and >40 indicate good quality.

### 2.4 Statistical analysis

Logit transformed prevalence data were used to calculate the pooled prevalence with 95% confidence interval (CI).^15^ Heterogeneity was assessed using Q test and *I* ^2^ statistics, *I* ^2^ values of 0%, 1–25%, 26–50%, 51–75%, and >75% were regarded as none, low, moderate, substantial, and considerable heterogeneity, respectively. Random‐effect models were used if any heterogeneity was observed. Publication bias was evaluated by funnel plots. Egger's test was performed to assess the funnel plot asymmetry when at least 10 studies were analysed. Subgroup analyses were conducted as follows: primary tumours or metastases, de novo NEPC (no prior diagnosis or treatment for prostate adenocarcinoma at the time of NEPC diagnosis) or t‐NEPC (with prior ADT for previous prostate adenocarcinoma), pure NEPC (pure small/large‐cell carcinoma) or mixed NEPC (small/large‐cell carcinoma mixed with prostate adenocarcinoma or prostate adenocarcinoma with neuroendocrine differentiation), and tissue‐based sequencing or liquid biopsies. The differences in genomic alteration prevalence between subgroups were evaluated using *Z* test. Statistical significance was set as *p* < 0.05. Meta‐analysis was conducted using R Studio (version 2022.02.0) with *meta* package (version 4.20‐2).

## 3 RESULTS

### 3.1 Study selection

**FIGURE 1:**
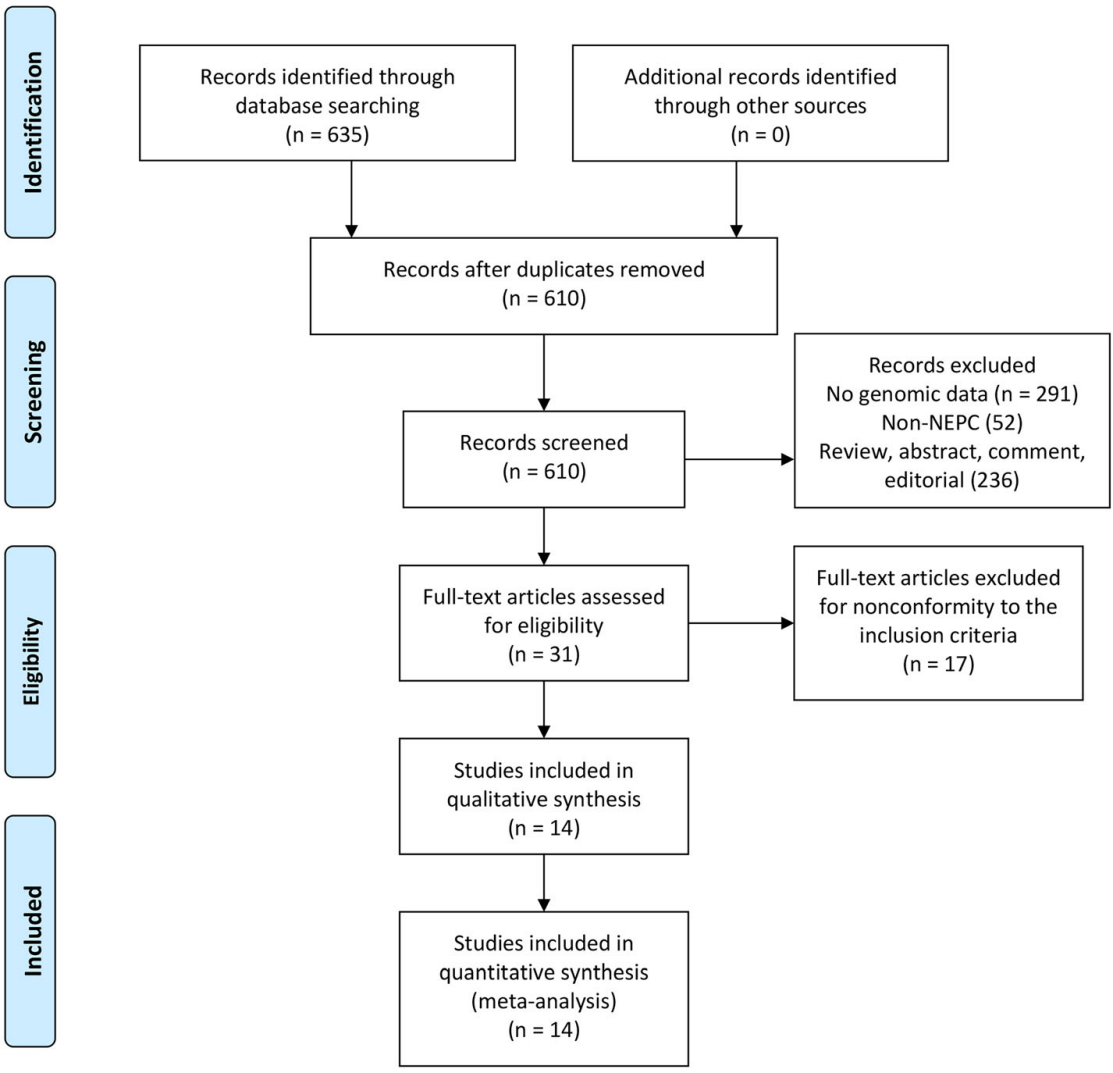
PRISMA flow diagram of study screening and selection

A total of 610 titles and abstracts were identified from PubMed, EMBASE, and Cochrane Central Register of Controlled Trials after the removal of duplicates. Of these, 579 records were excluded for not meeting the inclusion criteria, and the remaining 31 studies were further retrieved for full‐text screening. Finally, after a more careful selection, 14 articles were included in the systematic review and meta‐analysis. The detailed selection process is shown in Figure 1.

### 3.2 Study characteristics and quality assessment

The main characteristics of the eligible studies are shown in Table 1. Overall, 14 studies with 449 patients were included in the meta‐analysis.^3^, ^8^, ^9^, ^10^, ^16^, ^17^, ^18^, ^19^, ^20^, ^21^, ^22^, ^23^, ^24^, ^25^ The median number of patients enrolled in each study was 17 (range: 5–218), and the median age ranged from 65 to 77 years. Eleven studies performed gene sequencing on tumour tissues, two studies conducted liquid biopsies (circulating tumour cells and cell‐free DNA), and one study did not report the source of tumour DNA. Several methodologies were used to assess gene alterations, including single‐nucleotide polymorphism (SNP) array, fluorescence in situ hybridization (FISH), target next‐generation sequencing (NGS), whole‐exome sequencing (WES), and sanger sequencing.

**TABLE 1:** The main characteristics of the studies included in the meta‐analysis

Study	No. of patient^a^	Age, median (range)	De novo NEPC	Treatment‐emerging NEPC	Pure NEPC	Mixed NEPC	Samples	Primary tumour	Metastasis	DNA source	Methodology
Beltran 2011	7 37	NR	NR	NR	4 NR	3 NR	7 37	1 NR	5 NR	Frozen tissue FFPE tissue	SNP Array FISH
Tan 2014	13	NR	NR	NR	NR	NR	13	13	0	FFPE tissue	Target NGS Sanger sequencing
Beltran 2016	30	69 (55–87)	NR	NR	12	18	44	19	25	Frozen/FFPE tissue	WES
Aggarwal 2018	12	69 (55–90)^#^	0	12	8	4	12	0	12	FFPE tissue	Target NGS
Chedgy 2018	17	65 (43–78)	17	0	10	7	22	22	0	FFPE tissue	Target NGS/WES
Beltran 2019^b^	12	67 (45–87)^#^	NR	NR	12	0	12	0	12	Frozen tissue	WES
Abida 2019^c^	22	67 (49–87)	NR	NR	10	12	22	1	21	Frozen tissue	WES
Conteduca 2019^d^	47	67 (57–75)	24	23	21	26	NR	NR	NR	Frozen/FFPE tissue	WES
Beltran 2020	17	68 (54–90)	NR	NR	NR	NR	17	NR	NR	cfDNA	WES
Conteduca 2021‐CTC	7	70 (59–79)	2	5	2	5	7	NR	NR	CTC	NGS (not specified)
Jardim 2021	218	NR	NR	NR	NR	NR	NR	NR	NR	NR	Target NGS
Xiao 2021	5	65 (53–75)	NR	NR	5	0	5	3	2	FFPE tissue	NGS (not specified)
Zhu 2022	43	66 (57–78)	0	43	NR	NR	43	5	38	Frozen/FFPE tissue	Target NGS/WES
Ida 2022	9	77 (60–84)	8	1	2	7	9	9	0	FFPE tissue	Target NGS

Abbreviations: cfDNA, cell free DNA; CTC, circulating tumour cell; FFPE, formalin‐fixed paraffin‐embedded; NEPC, neuroendocrine prostate cancer; NGS, next‐generation sequencing; NR, not reported; WES, whole‐exome sequencing.

^a^ For studies involving both NEPC and prostate adenocarcinoma, only data of NEPC patients with sequencing data are included.

^b^ Excluding patients in Beltran 2016.

^c^ Excluding patients in Beltran 2016 and Beltran 2019.

^d^ Including patients in Beltran 2016, Beltran 2019, and Abida 2019.

Quality assessments were performed on the included studies (details summarized in Table S1). Seven studies were considered good quality, five studies were of moderate quality, and two studies were of poor quality.

### 3.3 Frequent gene mutations in NEPC

**FIGURE 2:**
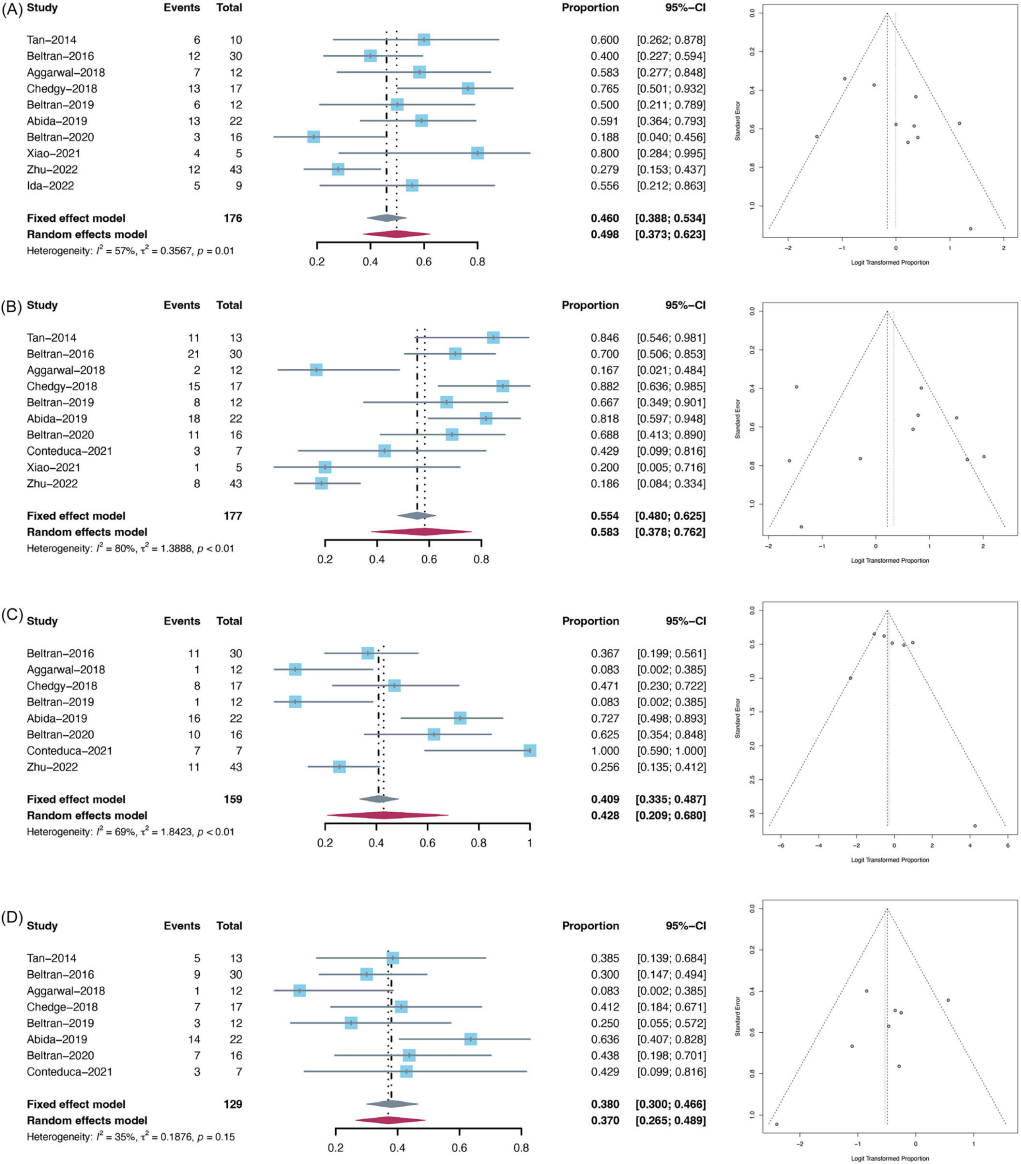
Forest plots (left) and funnel plots (right) of frequent mutations and copy number alterations in NEPC. (A) Pooled prevalence of *TP53* mutations; (B) pooled prevalence of *RB1* loss; (C) pooled prevalence of *TP53* loss; (D) pooled prevalence of *PTEN* loss

Among various gene mutations in NEPC, *TP53* was the most frequently mutated gene in the overall NEPC population (Table 2). A total of 10 studies reported the mutation prevalence of *TP53* in NEPC, ranging from 18.8% to 80%. The overall pooled prevalence of *TP53* mutation was 49.8% (95% CI: 37.3–62.3%, *I* ^2^ = 57%; Figure 2A). The funnel plot and Egger's test (*p* = 0.118) indicate a low likelihood of publication bias. Other frequent somatic mutations (prevalence >10%) in NEPC included *TTN*, *DST*, *MUC16*, *ZFHX4*, *ZNF479*, *CACNA1B*, *ZNF99*, *CMYA5*, *RYR1*, *OBSCN*, *KMT2D*, and *RYR2* (Table 2).

**TABLE 2:** Pooled prevalence of frequent somatic mutations in NEPC

Gene	Studies (no.)	Sample size	Pooled prevalence, % (95% CI)	*I* ^2^, %	Het. *p*
*TP53*	10	176	49.8 (37.3–62.3)	57	0.01
*TTN*	2	52	26.9 (16.6–40.5)	31	0.23
*DST*	1	30	23.3 (9.9–42.3)	‐	‐
*MUC16*	2	52	19.2 (10.7–32.2)	0	0.39
*ZFHX4*	1	30	13.3 (3.8–30.7)	‐	‐
*ZNF479*	1	30	13.3 (3.8–30.7)	‐	‐
*CACNA1B*	1	30	13.3 (3.8–30.7)	‐	‐
*ZNF99*	2	52	12.7 (4.7–29.9)	54	0.14
*CMYA5*	2	52	11.5 (5.3–23.4)	37	0.21
*RYR1*	2	52	11.5 (5.3–23.4)	0	0.64
*OBSCN*	2	52	11.5 (5.3–23.4)	0	0.69
*KMT2D*	2	52	11.5 (5.3–23.4)	0	0.69
*RYR2*	2	52	11.5 (5.3–23.4)	0	0.69

*Note*: Het = heterogeneity.

### 3.4 Copy number alterations in NEPC

CNAs of interest include *RB1* loss, *TP53* loss, and *PTEN* loss because of their critical roles in facilitating NEPC development.^11^, ^12^, ^26^ The prevalence of *RB1* loss was reported in 10 studies, ranging from 16.7% to 88.2%, and the overall pooled prevalence was 58.3% (95% CI: 37.8–76.2%, *I* ^2^ = 80%; Figure 2B) without obvious publication bias. The pooled prevalence of *TP53* loss and *PTEN* loss were 42.8% (95% CI: 20.9–68.0%, *I* ^2^ = 69%; Figure 2C) and 37.0% (95% CI: 26.5–48.9%, *I* ^2^ = 35%; Figure 2D), respectively.

When both gene mutations and CNAs were taken into consideration, the overall pooled prevalence of *RB1* alteration, *TP53* alteration, *RB1/TP53* alterations, and concurrent *RB1* and *TP53* alterations in NEPC were 64.2% (95% CI: 46.7–78.6%, *I* ^2^ = 78%), 65.5% (95% CI: 53.6–75.8%, *I* ^2^ = 45%), 83.8% (95% CI: 71.3–91.5%, *I* ^2^ = 52%), and 43.9% (95% CI: 28.2–60.9%, *I* ^2^ = 73%), respectively (Table 3).

**TABLE 3:** Pooled prevalence of common copy number alterations and potentially actionable alterations in NEPC

Gene	Studies (no.)	Sample size	Pooled prevalence, % (95% CI)	*I* ^2^, %	Het. *p*
*RB1* loss	10	177	48.3 (37.8–76.2)	80	<0.01
*TP53* loss	8	159	42.8 (20.9–68.0)	69	<0.01
*PTEN* loss	8	129	37.0 (26.5–48.9)	35	0.15
*RB1* alt	11	395	62.6 (46.2–76.6)	76	<0.01
*TP53 alt*	11	183	65.5 (3.8–30.7)	45	0.05
*RB1/TP53* alt	10	177	83.8 (71.3–91.5)	52	0.03
Concurrent *RB1 + TP53* alt	10	174	43.9 (28.2–60.9)	73	<0.01
*AURKA* amp	2	66	28.2 (11.5–54.3)	81	0.02
*MYCN* amp	2	59	22.9 (7.0–53.8)	82	0.02
del *ATM* alt	4	113	4.4 (1.9–10.2)	0	1.00
del *BRCA1* alt	4	113	2.7 (0.7–7.9)	0	0.90
del *BRCA2* alt	4	113	10.6 (6.1–17.8)	0	0.69
del *ATM/BRCA* alt	4	113	16.8 (11.0–24.9)	0	0.48

*Note*: Het = heterogeneity; alt = alteration, including mutation and copy number alteration; amp = amplification; del = deleterious.

### 3.5 Potentially actionable gene alterations in NEPC

Several recent clinical trials suggest that patients with mCRPC harbouring deleterious homologous recombination repair (HRR) gene alterations such as *BRCA1/*2 and *ATM* may benefit from PARP inhibitor‐based therapies.^27^, ^28^, ^29^ Furthermore, a phase 2 study indicated that amplification of *AURKA* and/or *MYCN* in prostate cancer conferred sensitivity to Aurora kinase A inhibition.^17^ Thus, we assessed the prevalence of these potentially actionable gene alterations in NEPC. Overall, four studies reported the prevalence of deleterious *BRCA/ATM* alterations. The pooled prevalence of deleterious *BRCA1*, *BRCA2*, *ATM* and *BRCA/ATM* alterations in NEPC were 2.7% (95% CI: 0.9–7.9%, *I* ^2^ = 0%), 10.6% (95% CI: 6.1–17.8%, *I* ^2^ = 0%), 4.4% (95% CI: 1.9–10.2%, *I* ^2^ = 0%), and 16.8% (95% CI: 11.0%–24.9%, *I* ^2^ = 0%), respectively (Table 3). Only two studies reported the prevalence of *AURKA* and *MYCN* amplifications in NEPC, and the results were highly variable. The overall pooled prevalence of *AURKA* and *MYCN* amplifications were 28.2% (95% CI: 11.5–54.3%, *I* ^2^ = 81%) and 22.9% (95% CI: 7.0–53.8%, *I* ^2^ = 82%), respectively (Table 3).

### 3.6 Comparison of genomic alterations between NEPC and advanced prostate adenocarcinoma

To further compare the prevalence of genomic alterations between NEPC and advanced prostate adenocarcinoma, we performed comparative analyses using our pooled results and the gene alteration frequency data in patients with mCRPC from the SU2C‐PCF dataset.^30^ Overall, there was a significantly higher prevalence of *RB1* alterations, *TP53* alterations, *AURKA* amplifications, and *MYCN* amplifications in NEPC compared with advanced prostate adenocarcinoma. However, the frequencies of *PTEN* loss and deleterious *ATM/BRCA* alterations were similar between the two groups (Figure S1).

### 3.7 Genomic alterations in different NEPC subgroups

**FIGURE 3:**
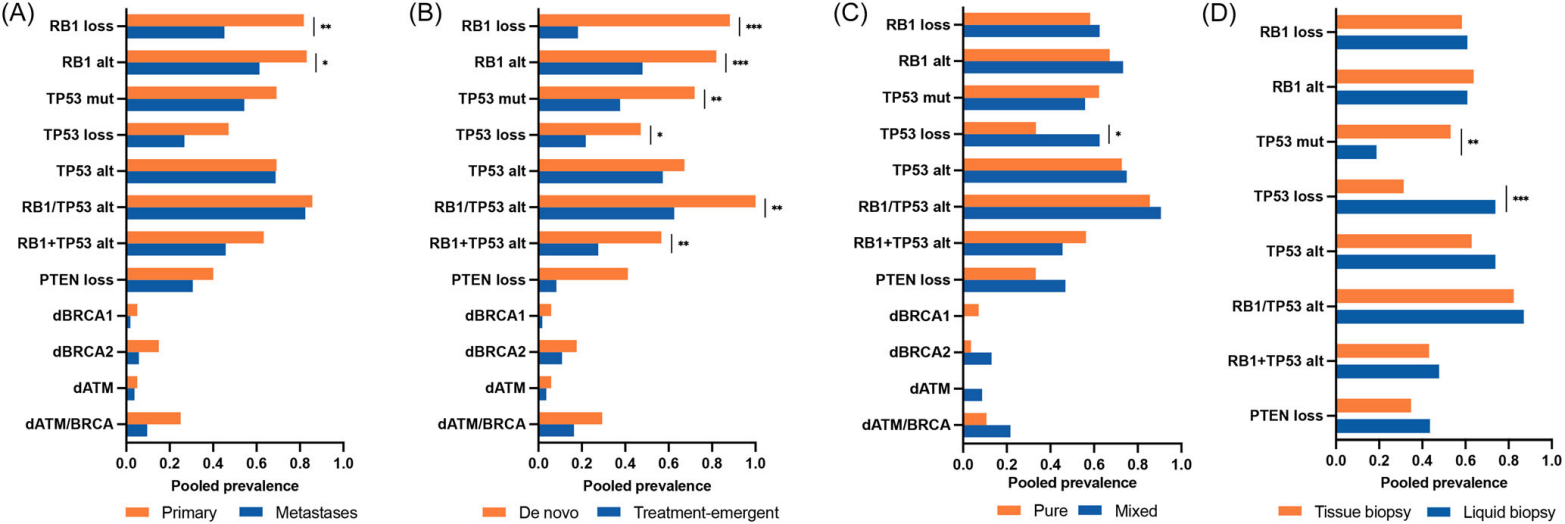
Pooled prevalence of selected genomic alterations comparing (A) primary tumours and metastases; (B) de novo NEPC and treatment‐emergent NEPC; (C) pure NEPC and mixed NEPC; and (D) liquid biopsy and tissue biopsy. *p < 0.05; **p < 0.01; ***p < 0.001

The prevalence of genomic alterations in different NEPC subgroups and their comparisons are presented in Figure 3. Compared with metastases, primary NEPC tumours showd more genomic alterations in *RB1* (83.1% vs. 61.4%, *p* = 0.044) but not in any other commonly altered genes. Interestingly, the differences in genomic alterations between de novo NEPC and t‐NEPC were more significant. De novo NEPC had a higher prevalence of *RB1*, *RB1/TP53*, and concurrent *RB1* and *TP53* alterations than t‐NEPC (82.0%, 100.0%, and 56.6% in de novo NEPC compared with 48.0%, 62.5%, and 27.5% in t‐NEPC, respectively). Although the prevalence of *TP53* loss was higher in mixed NEPC than in pure NEPC, no difference in the prevalence of overall *TP53* genomic alterations was observed. In addition, we also compared the detection rates between tissue‐based sequencing and liquid biopsies. We found that except for *TP53* mutation and *TP53* loss, the prevalence of other common genomic alterations was similar between tissue‐based sequencing and liquid biopsies.

## 4 DISCUSSION

To our knowledge, this is the first systematic review and meta‐analysis evaluating the prevalence of genomic alterations in NEPC. In this study, we reported more reliable prevalence data on gene mutations and CNAs in NEPC and confirmed the pervasiveness of *TP53* and *RB1* aberrations in this malignancy. Furthermore, we assessed potentially actionable gene alterations to better inform precision medicine and revealed the genomic differences between de novo NEPC and t‐NEPC.

Based on our pooled results, the most frequently altered genes in NEPC were *TP53*, *RB1*, and *PTEN*. These three tumour suppressors are also commonly inactivated in advanced prostate adenocarcinoma, especially in mCRPC.^8^, ^30^, ^31^ Comparing our pooled results with data from the SU2C‐PCF dataset, the prevalence of *PTEN* loss in NEPC was similar to that in advanced prostate adenocarcinoma, while *RB1* and *TP53* were more frequently altered in NEPC.^30^ Recent studies with PCa cell lines and genetically engineered mouse models indicate that combined, but not single, knockdown of *RB1* and *TP53* can induce NE transdifferentiation.^11^, ^12^ Another previous study found that combined inactivation of *TP53* and *PTEN* in PCa could lead to abiraterone resistance and the acquisition of NE phenotype.^26^ In addition, triple deletion of *RB1*, *TP53*, and *PTEN* in mouse models promotes aggressive PCa and lineage plasticity.^11^ These three genes reportedly induce NE transdifferentiation via upregulating stemness/NE program‐related transcription factors and epigenetic regulators, such as *SOX2*, *SOX11*, and *EZH2*.^11^, ^12^, ^26^ However, a more recent study with LNCaP cells and patient‐derived xenograft (PDX) models shows that combined deletion of *TP53* and *RB1* does not obligate the acquisition of NE phenotype, suggesting the involvement of other molecular events during NE transdifferetiation in PCa.^32^ Our meta‐analysis identified 12 additional genes with a mutation prevalence of >10% in NEPC but only from a limited number of studies. Although the exact roles of these genes in NEPC development remain to be elucidated, some of them (i.e., TTN, MUC16, and KMT2D) are reportedly associated with disease progression and treatment resistance in prostate adenocarcinoma and other types of cancers.^33^, ^34^, ^35^, ^36^, ^37^, ^38^, ^39^

Genomic interrogation not only helps us understand the mechanisms underlying NEPC development and aggressiveness but also facilitates precision medicine. Alisertib is a small molecule inhibitor of Aurora kinase A that disrupts the N‐myc‐Aurora A protein complex, thus inhibiting the mitotic process and tumour growth.^40^, ^41^ A phase 2 study has evaluated the efficacy of alisertib treatment in NEPC.^17^ Although the study failed to meet its primary endpoint, it demonstrated an association between *AURKA* amplification and improved survival. It also identified exceptional responders with MYCN overactivity. According to our results, the pooled prevalence of *AURKA* and *MYCN* amplifications in NEPC were only 28.2% and 22.9%, respectively. However, the proportion of patients with AURKA/MYCN overexpression in NEPC is higher than that of gene amplification, and these patients may also benefit from alisertib treatment.^9^, ^17^ Recently, several large clinical trials have demonstrated the effectiveness of PARP inhibitors in mCRPC patients with HRR defects, especially in those harbouring deleterious *ATM* or *BRCA1/2* mutations.^27^, ^28^, ^29^ In addition, a retrospective study showed that DNA‐repair genes status was associated with treatment response and progression‐free survival in t‐NEPC patients receiving platinum‐based chemotherapy.^25^ In our study, we focused on the deleterious mutations in *ATM* and *BRCA1/2* because of their strong therapeutic predictive value in PCa. The overall pooled prevalence of deleterious *ATM/BRCA* mutations in NEPC was 16.8%, with *BRCA2* mutations being the most frequent at 10.6%. This is a similar mutation profile to advanced prostate adenocarcinoma.^30^, ^31^ Taken together, these findings strongly suggest that genomic testing should be performed in NEPC patients to help decide appropriate treatments.

Although t‐NEPC is more commonly observed, a small proportion of patients can present with de novo NEPC. Given the rarity of de novo NEPC, whether it is molecularly distinct from t‐NEPC remains unclear. From the perspective of genomic alterations, we observed a significantly higher prevalence of concurrent *RB1/TP53* alterations and numerically more *PTEN* loss and *ATM/BRCA* mutations in de novo NEPC than in t‐NEPC. Based on these findings, it is reasonable to speculate that tumours with more intrinsic genomic alterations, especially with driver events such as *RB1/TP53* alterations, are more likely to evolve towards an NE phenotype even in the absence of therapeutic stress. Other tumours may acquire these same genomic changes during the course of therapy or undergo epigenetic processes to facilitate lineage plasticity, ultimately becoming t‐NEPC. In support of this speculation, a recent study on matched pre‐ and post‐NEPC samples demonstrated that *RB1* alterations in post‐NEPC samples were only detected in a minority of matched pre‐NEPC samples.^42^ We previously established a unique PDX model of prostate adenocarcinoma (LTL331) transdifferentiation into NEPC (LTL331R) following castration.^43^ In this system, the LTL331 and LTL331R models share remarkably similar genomic profiles, and both harbour a single‐copy loss of *RB1* and *TP53*. However, transcriptomic data showed a higher *RB1* loss signature in LTL331R than in LTL331, suggesting that the *RB1* pathway is not fully inactivated in LTL331. As such, epigenetic dysregulation may facilitate genomic changes to promote NE transdifferentiation in t‐NEPC.^44^ Data from genetically engineered mouse models indicate that an NE phenotype driven by either MYCN overexpression or *RB1* loss can also exhibit increased expression of epigenetic reprogramming factors such as EZH2.^11^, ^45^ In addition, whole‐genome bisulfite sequencing also reveals marked differences in DNA methylation between NEPC and CRPC.^8^ Thus, future studies are needed to unveil whether de novo NEPC and t‐NEPC are epigenetically different.

The genomic changes in pure and mixed NEPC appear most consistent. There are also numerically more genomic alterations in NEPC metastases than in primary NEPC tumours. However, these results should be interpreted cautiously. Many of the primary tumours sequenced in the relevant studies were also de novo NEPC, which may be a confounding factor due to their higher frequency of genomic changes. In addition, there is a lack of data on paired primary and metastatic tumours, making it challenging to explore the association between genomic aberrations and drivers of metastasis in NEPC. Furthermore, we also compared the detection rates between liquid biopsies and tissue‐based sequencing. We found that, except for *TP53* mutations and *TP53* losses, there was no significant difference between these two methods. Notably, a recent study in NEPC patients detecting genomic alterations in matched plasma and tissue samples found a high concordance between cell‐free DNA and biopsy tissues.^18^ Therefore, liquid biopsy can be a promising supplement to tissue‐based sequencing in NEPC.

Indeed, our study has several limitations. Due to difficulties in diagnosing NEPC and limited metastases biopsies, most included studies are relatively small, resulting in a modest sample size for this meta‐analysis. In some of the eligible studies, individual sequencing data were not available and complete clinical information was scarce, leading to fewer patients in the subgroup analyses. Additionally, for the comparisons between primary and metastatic tumours, and between liquid and tissue biopsies, the data used for analysis were not generated from paired samples, thereby limiting the interpretation of the results. Finally, due to limited data on whole‐genome changes in NEPC, we mainly focused on the most frequent genomic alterations. Other less common but potentially biologically critical gene aberrations could be missed in this meta‐analysis.

## 5 CONCLUSIONS

This meta‐analysis provides the most currently comprehensive prevalence of genomic alterations in NEPC. Our results confirm pervasive *RB1* and *TP53* alterations in NEPC. We also present the frequency of potentially actionable mutations, highlighting that genomic testing should be performed in NEPC patients to select candidates for precision medicine. Finally, our analyses reveal the genomic differences between de novo NEPC and t‐NEPC, provide insights for future studies and molecular characterizations of different NEPCs.

## CONFLICT OF INTEREST

The authors declare that they have no known competing financial interests or personal relationships that could have appeared to influence the work reported in this paper.

## AUTHOR CONTRIBUTIONS

Conceptualization: JC, MS, HZ and YZW; Methodology: JC, MS; Data Analysis and Interpretation: JC, MS, YW, DL; Writing: JC, MS, SC,YW, DL; Review & Editing: SC, HZ, YZW; Fubding: HZ, YZW.

## Supporting information


**Figure S1.** Comparison of the prevalence of selected genomic alterations between NEPC and metastatic castration‐resistant prostate adenocarcinoma. *p < 0.05; **p < 0.01; ***p < 0.001.

**Table S1.** Quality assessment of included studies via Q‐genie tool
