# Supplementary figures and images for: Genomic alterations in neuroendocrine prostate cancer: A systematic review and meta‐analysis
Source: BJUI Compass. 2023 Jan 2;4(3):256–65. doi: 10.1002/bco2.212 (PMC10071089; doi:10.1002/bco2.212)

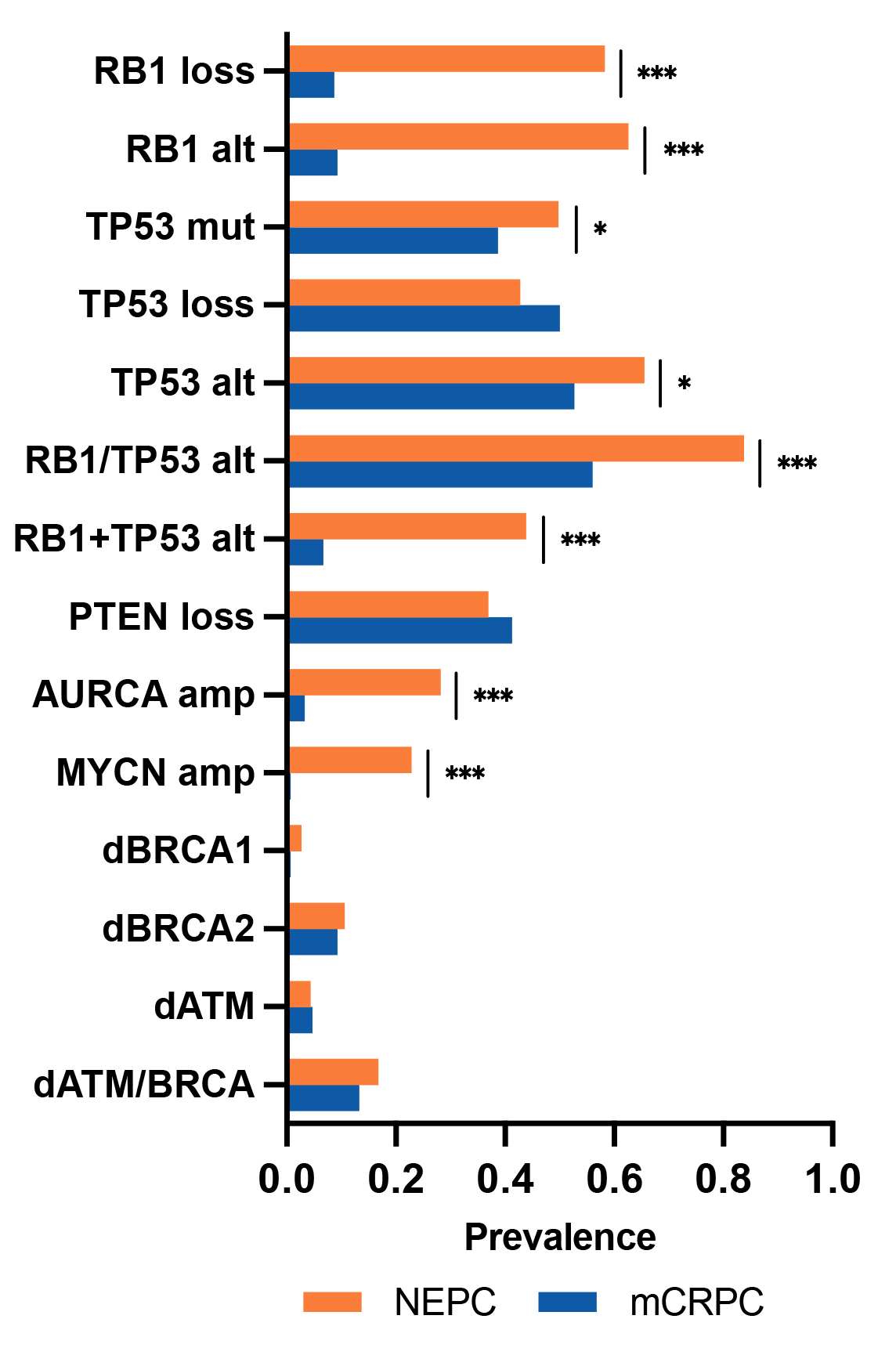

Supplement: Supplementary file 1 — Figure S1. Comparison of the prevalence of selected genomic alterations between NEPC and metastatic castration‐resistant prostate adenocarcinoma. *p < 0.05; **p < 0.01; ***p < 0.001. [file BCO2-4-256-s002.jpg]
