# Supplementary material for: Genomic alterations in neuroendocrine prostate cancer: A systematic review and meta‐analysis
Source: BJUI Compass. 2023 Jan 2;4(3):256–65. doi: 10.1002/bco2.212 (PMC10071089; doi:10.1002/bco2.212)
Supplement: Supplementary file 2 — Table S1. Quality assessment of included studies via Q‐genie tool [file BCO2-4-256-s001.docx]

Table S1. Quality assessment of included studies via Q-genie tool

| Study | Rationale for study | Selection and outcome | Comparability of comparison group | Technical exposure | Non-technical exposure | Other sources of bias | Sample size and power | A priori planning of analysis | Statistical methods and control for confounding | Testing of assumptions and inferences for genetic analysis | Appropriateness of inferences drawn from results | Score |
| --- | --- | --- | --- | --- | --- | --- | --- | --- | --- | --- | --- | --- |
| Beltran 2011 | 6 | 5 | 1 | 3 | 2 | 3 | 3 | 4 | 2 | 5 | 6 | 40 |
| Tan 2014 | 6 | 4 | 1 | 3 | 2 | 2 | 3 | 2 | 1 | 4 | 5 | 33 |
| Beltran 2016 | 6 | 5 | 1 | 5 | 2 | 3 | 3 | 5 | 2 | 5 | 5 | 42 |
| Aggarwal 2018 | 6 | 6 | 5 | 3 | 4 | 4 | 3 | 6 | 2 | 5 | 6 | 50 |
| Chedge 2018 | 6 | 5 | 1 | 2 | 2 | 4 | 3 | 5 | 1 | 5 | 6 | 40 |
| Beltran 2019 | 6 | 5 | 1 | 3 | 2 | 4 | 6 | 6 | 1 | 5 | 5 | 44 |
| Abida 2019 | 6 | 6 | 1 | 3 | 2 | 4 | 3 | 6 | 2 | 5 | 6 | 44 |
| Conteduca 2019 | 6 | 5 | 5 | 3 | 2 | 4 | 4 | 6 | 2 | 5 | 6 | 48 |
| Beltran 2020 | 6 | 5 | 5 | 5 | 2 | 5 | 3 | 5 | 3 | 5 | 6 | 50 |
| Conteduca 2021 | 6 | 4 | 4 | 2 | 2 | 4 | 2 | 2 | 2 | 4 | 4 | 36 |
| Jardim 2021 | 6 | 1 | 1 | 1 | 1 | 1 | 4 | 2 | 1 | 4 | 4 | 26 |
| Xiao 2021 | 6 | 4 | 1 | 1 | 2 | 2 | 2 | 2 | 1 | 4 | 4 | 29 |
| Zhu 2022 | 6 | 5 | 5 | 4 | 2 | 4 | 3 | 5 | 2 | 4 | 6 | 46 |
| Ida 2022 | 6 | 5 | 1 | 2 | 2 | 4 | 2 | 2 | 1 | 4 | 4 | 33 |
